# A Novel Role for Brain Natriuretic Peptide: Inhibition of IL-1β Secretion via Downregulation of NF-kB/Erk 1/2 and NALP3/ASC/Caspase-1 Activation in Human THP-1 Monocyte

**DOI:** 10.1155/2017/5858315

**Published:** 2017-02-26

**Authors:** Letizia Mezzasoma, Cinzia Antognelli, Vincenzo Nicola Talesa

**Affiliations:** Department of Experimental Medicine, University of Perugia, Piazzale L. Severi 1, 06129 Perugia, Italy

## Abstract

Interleukin-1*β* (IL-1*β*) is a pleiotropic cytokine and a crucial mediator of inflammatory and immune responses. IL-1*β* processing and release are tightly controlled by complex pathways such as NF-kB/ERK1/2, to produce pro-IL-1*β*, and NALP3/ASC/Caspase-1 inflammasome, to produce the active secreted protein. Dysregulation of both IL-1*β* and its related pathways is involved in inflammatory/autoimmune disorders and in a wide range of other diseases. Identifying molecules modulating their expression is a crucial need to develop new therapeutic agents. IL-1*β* is a strong regulator of Brain Natriuretic Peptide (BNP), a hormone involved in cardiovascular homeostasis by guanylyl cyclase Natriuretic Peptide Receptor (NPR-1). An emerging role of BNP in inflammation and immunity, although proposed, remains largely unexplored. Here, we newly demonstrated that, in human THP-1 monocytes, LPS/ATP-induced IL-1*β* secretion is strongly inhibited by BNP/NPR-1/cGMP axis at all the molecular mechanisms that tightly control its production and release, NF-kB, ERK 1/2, and all the elements of NALP3/ASC/Caspase-1 inflammasome cascade, and that NALP3 inflammasome inhibition is directly related to BNP deregulatory effect on NF-kB/ERK 1/2 activation. Our findings reveal a novel potent anti-inflammatory and immunomodulatory role for BNP and open new alleys of investigation for a possible employment of this endogenous agent in the treatment of inflammatory/immune-related and IL-1*β*/NF-kB/ERK1/2/NALP3/ASC/Caspase-1-associated diseases.

## 1. Introduction

IL-1*β*, released by activated monocytes and macrophages, is a critical player in proinflammatory pathways activation in nearly every tissue and organ [1–3]. In particular, IL-1*β* is implicated in both acute and chronic inflammation and in cell growth, differentiation, angiogenesis, profibrogenic mediators production, immune response regulation, and carcinogenesis [1–11]. A fine-tune control of its secretion is required and achieved by a two-steps mechanism acting both at transcriptional and at posttranslational level. Firstly, the inactive cytoplasmic precursor (pro-IL-1*β*) is synthesized; subsequently the active protein is produced by Caspase-1 proteolytic cleavage of the precursor and released into the extracellular space where it initiates its wide range of responses [1–5, 12]. The first step of the mechanism requires the activation of specific complex pathways, such as nuclear factor-kB (NF-kB) and extracellular-signal regulated kinases 1/2 (ERK 1/2) [4, 6, 13]. The second step requires the activation of molecular platforms called inflammasomes, inducing the activation of the proteolytic enzyme Caspase-1. Inflammasomes are composed of nucleotide-binding domain leucine-rich repeat (NLR) receptors, adaptor proteins, and the effector protein pro-Caspase-1 [1–6]. The most extensively studied inflammasome is NLRP3/NALP3, formed by the NLR pyrin domain containing 3 (NLRP3, also known as NALP3 or cryopyrin) receptors and by the apoptosis-associated speck-like protein containing a Caspase activation and recruitment domain (ASC) adapter [1–6]. NALP3 inflammasome activation, induced by a large variety of endogenous and exogenous stimuli, appears to occur in two steps. The first one, priming/licensing step, leads to the activation of NF-KB-mediated signaling, which in turn upregulates transcription of inflammasome-related components, including inactive NALP3 and pro-IL-1*β*. The second one is the oligomerization of NALP3 and subsequent assembly of NALP3/ASC/pro-Caspase-1 to a complex triggering the activation of pro-Caspase-1 in Caspase-1 active fragment, as well as the production and secretion of mature IL-1*β*. Once activated, NALP3 represents a major player in innate immune host defense [2–4, 6, 14].

As known, NF-kB and ERK 1/2 pathways play a crucial role in a wide range of processes such as inflammation, stress response, apoptosis, cell survival, proliferation, angiogenesis, and innate and acquired immunity. In addition, their dysregulation is involved in a variety of diseases, including autoimmune, neurodegenerative, and inflammatory disorders, and in cancer [15–18]. Likewise, inappropriate activation of IL-1*β* and NALP3 is a common feature of a wide range of major human diseases, such as gout [19], type 2 diabetes [20], obesity-induced insulin resistance [21], Crohn's diseases [22], silicosis [23], psoriasis [10], cancer [4, 24], atherosclerosis [8], and Alzheimer's disease [25], known to affect millions of people worldwide. Finally, ASC itself is also involved in inflammasome-independent cascade: Caspase-1 independent activation of necrosis [26], antigens-induced arthritis [27], apoptosis in tumor cell lines [28], and regulation of inflammatory cytokines production, by NF-kB and MAPK pathways activation [2, 28]. Hence, the extensive involvement of IL-1*β*, NF-kB, ERK 1/2, NALP3, ASC and Caspase-1 in such a range of diseases makes them high desirable drug targets.

IL-1*β* is a strong regulator of Brain Natriuretic Peptide BNP (BNP) [29], a member of the Natriuretic Peptides (NPs) family, synthetized and secreted constitutively by the heart, deeply involved in body fluid and cardiovascular homeostasis [30, 31]. BNP is upregulated in myocardium in response to many pathophysiological stimuli and exerts its biological functions by guanylyl cyclase Natriuretic Peptide Receptor-1 (NPR-1/NPR-A) [30–34]. NPR-1 activation induces an increase of cGMP intracellular levels, leading to the activation of specific cGMP-dependent protein kinases, phosphodiesterases and cyclic nucleotide gated cation channels [30, 31, 34]. NPs and NPR-1 are expressed in many tissues and exert endocrine, autocrine, and paracrine effects in normal or pathological conditions [30, 31, 34] and a wide range of further activities, including an emerging role on inflammation and immunity [30, 31, 35–38]. In particular, BNP plasma level is upregulated in patients during inflammatory contexts, such as sepsis or septic shock [37, 39] and in several inflammatory diseases with or without cardiac dysfunction [37]. In addition, the main cytokines released during inflammation increase BNP production and secretion in vitro [37, 40, 41] and in many conditions where IL-1*β* and other cytokines levels are increased [42], BNP plasma levels may be particularly elevated [43]. Finally, BNP inhibits monocytes chemotaxis [35] and influences some inflammatory mediators production in macrophages [36] and NPR-1 is expressed on immune cells [30, 35, 38, 44, 45]. Altogether, these observations suggest a role for BNP in inflammation and its possible immunomodulatory action. However, the relationship between BNP/inflammation/immune systems still remains to be widely investigated. In addition, despite BNP modulates some proinflammatory cytokines production [36], no data exist about its effect on human IL-1*β* secretion.

The aim of our study was to evaluate BNP effect on IL-1*β* release and NF-kB, ERK 1/2, NALP3, ASC, and Caspase-1 activation in LPS/ATP stimulated human THP-1 monocytes. For the first time to our knowledge, we demonstrated that BNP/NPR-1/cGMP axis strongly downregulated IL-1*β* secretion from THP-1 monocytes by inhibiting NF-kB and ERK 1/2 activation and all the elements of NALP3 inflammasome cascade. Analyzing the mechanism of action of BNP-dependent NALP3 inflammasome inhibition, we demonstrated that it is directly related to BNP deregulatory effect on NF-kB/ERK 1/2 activation. Our data, demonstrating such multiple inhibitory actions of BNP, reveal a novel potent anti-inflammatory and immunomodulatory role for this peptide. In addition, our study suggests that BNP might be a promising therapeutic agent for the treatment of inflammatory/immune-related and IL-1*β*/NF-kB/ERK1/2/NALP3/ASC/Caspase-1-associated diseases and, in general, in those pathophysiological scenarios where inflammatory and immune networks are involved.

## 2. Materials and Methods

### 2.1. Reagents

All the chemicals used in the present study were analytical grade reagents from various sources. Human BNP was obtained from Phoenix Europe GmbH (Germany) and dissolved in H_2_O. LPS, ATP, and Phorbol 12-myristate 13-acetate (PMA) were purchased from Sigma-Aldrich (Italy) and dissolved in RPMI, H_2_O and DMSO, respectively. The NF-kB and ERK 1/2 inhibitors, BAY 11-7082, and U0-126, respectively, were obtained from Santa Cruz Biotechnology, Inc. (Germany) and dissolved in 0.5% DMSO. NALP3 mouse monoclonal antibody (mAb) was from Adipogen International (USA). Caspase-1 rabbit polyclonal antibody (pAb), Phospho-IkB-*α* (Ser32) rabbit mAb, Phospho-p44/42 MAPK (ERK 1/2) (Thr202/Tyr204) rabbit pAb, IL-1*β* mouse mAb, and the appropriate HRP-conjugated secondary Abs were from Cell Signaling Technology (USA). NPR-1 rabbit pAb and *β*-actin mouse mAb were from Santa Cruz Biotecnology, Inc. (Germany). ASC rabbit pAb was from Enzo Life Sciences (USA).

### 2.2. Cell Culture and Drug Treatments

Human THP-1 monocytes were purchased from American Type Culture Collection (ATCC, USA) and routinely maintained at 37°C in 5% CO_2_ in RPMI 1640 supplemented with 10% heat inactivated (1 h at 56°C) FBS, 1x L-glutamine, 1 mM sodium pyruvate, 1x nonessential amino acids, 100 units/mL of penicillin, and 0.1 mg/mL of streptomycin (Invitrogen, Italy). THP-1 cells were plated (2 × 10^6^ cells/well) in 35 mm six-well culture dishes and treated for 30 min or 24, 48, and 72 h with 10 *μ*g/mL LPS/5 mM ATP in the presence or absence of 10^−6^ and 10^-8 ^M human BNP. In independent experiments, 10 *μ*M BAY 11-7082 or U0-126 were added to cells 1 h before LPS/ATP treatment. In independent experiments, THP-1 cells were differentiated to macrophage-like cells by 20 nM PMA for 72 h, washed three times with PBS, and plated (2 × 10^6^ cells/well) in 35 mm six-well culture dishes and, after 24 h, treated for 30 min with 10 *μ*g/mL LPS/5 mM ATP in the presence or absence of 10^-8 ^M human BNP. DMSO was used at 0.0005% assay concentration producing no significant toxicity. Control cells with DMSO did not show any significant difference with respect to control cells in RPMI 1640 medium; therefore all the relative treatments were compared to these latter controls.

### 2.3. Cell Viability

Cell viability was measured with a standard trypan blue uptake assay. Cell cultures were also morphologically examined via light microscopy.

### 2.4. Measurements of Secreted IL-1*β*

Measurements of secreted IL-1*β* were performed in 100 *μ*L supernatant after THP-1 exposure to the indicated compounds for 24, 48, and 72 h at 37°C. After treatments, supernatants were collected and human IL-1*β* levels were determined by the specific ELISA kit, according to the manufacturer's guidelines (eBioscience, USA).

### 2.5. cGMP Measurements

cGMP intracellular levels were determined by the specific EIA kit, according to the manufacturer's guidelines (GE Healthcare, UK). THP-1 cells were plated (1 × 10^6^ cells/well) in 48-well culture dishes, incubated for 5 and 30 min with 10^−8^ M human BNP, in absence or presence of 10 ug/mL LPS/5 mM ATP, and lysed in 200 *μ*L of the provided lysis buffer. Measurements were performed in 50 *μ*L of the lysate.

### 2.6. Western Blot Analysis

Total proteins (20 *μ*g) were separated by 12% sodium dodecyl sulfate-polyacrylamide gel electrophoresis (SDS-PAGE) and blotted onto a nitrocellulose membrane, using iBlot Dry Blotting System (Invitrogen, Italy). Nonspecific binding sites were blocked in Roti-Block (Roth GmbH, Germany) for 1 h at room temperature. The membranes were blotted overnight at 4°C with Roti-Block containing the following anti-human Abs : NPR-1 (1 : 200 dilution), ASC (1 *μ*g/mL), NALP3, Caspase-1, Phospho-IkB-*α*, Phospho-p44/42 MAPK, and IL-1*β* (1 : 1000 dilution). After washing with TBST, antigen-Ab complexes were detected by incubation for 1 h at room temperature with the appropriated HRP-conjugated secondary Abs (1 : 2000 dilution) and revealed using the enhanced chemiluminescence (ECL) system by Amersham Pharmacia, Biotech (Sweden). As internal loading controls and for normalizing purpose, all membranes were stripped of the first antibody and reprobed with anti-*β*-actin Ab (1 : 1000 dilution). Densitometry analyses were performed with ImageJ software.

### 2.7. Statistical Analysis

Results were expressed as means ± SD of three independent experiments performed in triplicate. The statistical significance of differences between treated and untreated cells was assessed by Student's *t*-test. Differences between groups were considered significant when *P* < 0.05.

## 3. Results

### 3.1. BNP Decreases LPS/ATP-Induced IL-1*β* Release in THP-1 Cells

**Figure 1:**
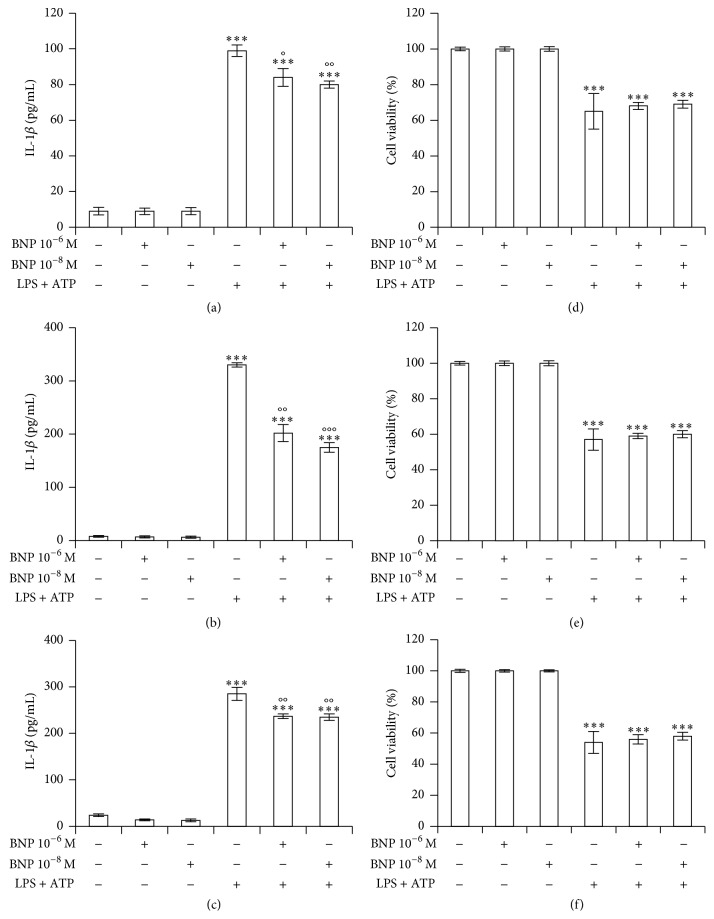
BNP decreases LPS/ATP-induced IL-1*β* release in THP-1 cells. THP-1 cells were treated with 10^−6^ and 10^−8 ^M BNP in absence or presence of 10 *μ*g/mL LPS/5 mM ATP for 24 (a, d), 48 (b, e), and 72 h (c, f). The supernatants were collected and IL-1*β* release was measured by ELISA (a, b, c). Cell viability was assessed by trypan blue uptake assay (d, e, f). Histograms indicate means ± SD of three separate experiments each one tested in triplicate. ^*∗∗∗*^*P* < 0.001 versus untreated cells and °*P* < 0.05, °°*P* < 0.01, and °°°*P* < 0.001 versus LPS/ATP treated cells.

We firstly evaluated the effect of 10^−6^ and 10^-8 ^M BNP on LPS/ATP-induced IL-1*β* release in THP-1 culture medium after 24 (Figure 1(a)), 48 (Figure 1(b)) and 72 h (Figure 1(c)) cotreatment. We demonstrated that BNP inhibited LPS/ATP-induced IL-1*β* secretion at all doses and exposure times, the effect obtained being already at 10^-8 ^M after 48 h treatment. BNP did not affect cell viability at any concentrations used (Figures 1(d), 1(e), and 1(f)). On the contrary, LPS/ATP treatment induced a marked cell death at all the exposure times (Figures 1(d), 1(e), and 1(f)), thus suggesting an intense NALP3 inflammasome activation.

### 3.2. BNP Activates NPR-1 in THP-1 Cells

**Figure 2:**
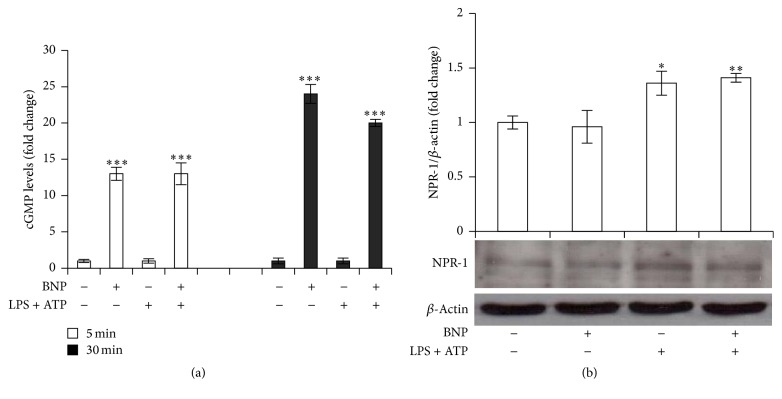
BNP activates NPR-1 in THP-1 cells. (a) THP-1 cells were treated with 10^−8 ^M BNP in absence or presence of 10 *μ*g/mL LPS/5 mM ATP and cGMP intracellular levels were evaluated after 5 and 30 min treatment. (b) THP-1 cells were treated with 10^−8 ^M BNP in absence or presence of 10 *μ*g/mL LPS/5 mM ATP for 30 min. Cell lysates were immunoblotted for NPR-1. The blots were stripped of the bound Ab and reprobed with mouse anti-*β*-actin, to confirm equal loading. Western blots are representative of three separate experiments. Histograms indicate means ± SD of three separate experiments each one tested in triplicate. ^*∗*^*P* < 0.05, ^*∗∗*^*P* < 0.01, and ^*∗∗∗*^*P* < 0.001 versus untreated cells.

BNP promotes its biological effects binding to NPR-1 [30, 31, 34] that, once activated, induces an increase in cGMP intracellular levels. We showed that 10^-8 ^M BNP treatment produced a significant increase in cGMP intracellular levels, either after 5 min or 30 min exposure (Figure 2(a)), without affecting NPR-1 protein expression (Figure 2(b)), thus indicating BNP/NPR-1/cGMP pathway activation.

### 3.3. BNP Is Involved in the Priming Mechanism Controlling IL-1*β* Production by Inhibiting NF-kB and ERK 1/2 Activation in THP-1 Cells

**Figure 3:**
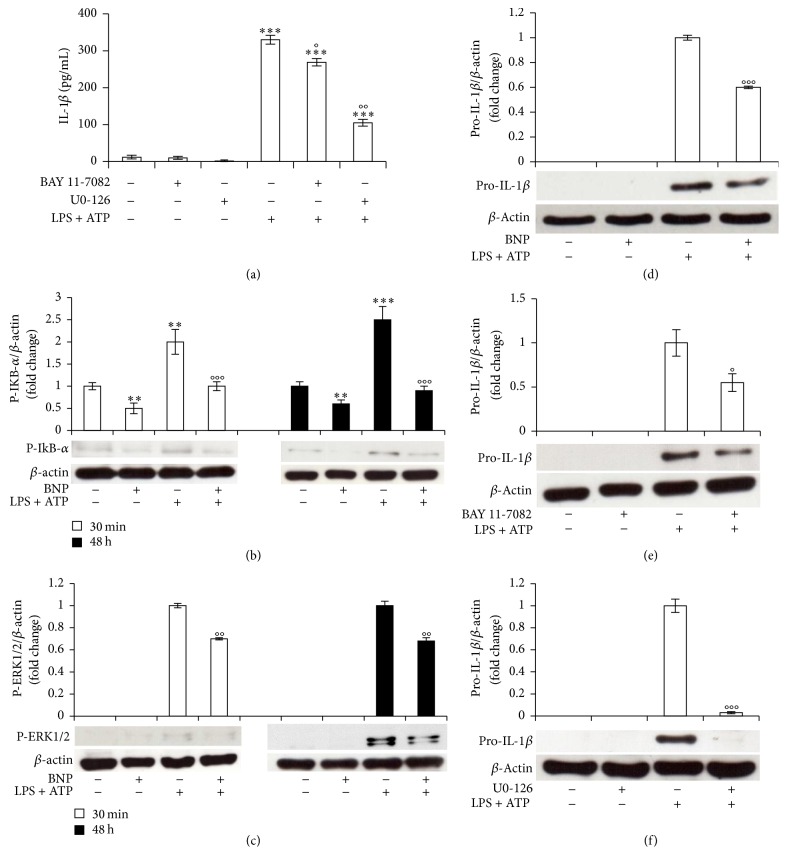
BNP is involved in the priming mechanism controlling IL-1*β* production by inhibiting NF-kB and ERK 1/2 activation in THP-1 cells. (a) THP-1 cells were incubated with 10 *μ*M BAY 11-7082 or U0-126 for 1 h and then treated with 10 *μ*g/mL LPS/5 mM ATP for 48 h. The supernatants were collected and IL-1*β* release was measured by ELISA. (b, c, d) THP-1 cells were treated with 10^−8 ^M BNP in absence or presence of 10 *μ*g/mL LPS/5 mM ATP for 30 min (b, c) or 48 h (b, c, d). Cell lysates were immunoblotted for P-IkB-*α* (b), P-ERK 1/2 (c), or pro-IL-1*β* (d). (e, f) THP-1 cells were incubated with 10 *μ*M BAY 11-7082 (e) or U0-126 (f) for 1 h and then treated with 10 *μ*g/mL LPS/5 mM ATP for 48 h. Cell lysates were immunoblotted for pro-IL-1*β*. The blots were stripped of the bound Ab and reprobed with mouse anti-*β*-actin, to confirm equal loading. Western blots are representative of three separate experiments. All histograms indicate means ± SD of three separate experiments each one tested in triplicate. ^*∗∗*^*P* < 0.01 and ^*∗∗∗*^*P* < 0.001 versus untreated cells and °*P* < 0.05, °°*P* < 0.01, and °°°*P* < 0.001 versus LPS/ATP treated cells.

IL-1*β* secretion is tightly controlled by two signals, one inducing the upregulation of the intracellular pro-IL-1*β* and the other one driving the secretion of the active molecule. LPS/ATP-induced inflammatory mediators production being predominantly regulated by NF-kB and ERK1/2 signaling [6, 15, 46], we firstly demonstrated the effective involvement of these pathways in our system by using the specific NF-kB and ERK 1/2 inhibitors BAY 11-7082 and U0-126, respectively. In fact, 10 *μ*M BAY 11-7082 or U0-126 1 h pretreatment reduced 48 h LPS/ATP-induced IL-1*β* release (Figure 3(a)). We then demonstrated that 10^-8 ^M BNP treatment abrogated and downregulated LPS/ATP-induced NF-kB and ERK 1/2 activation, respectively (Figures 3(b) and 3(c)), as indicated by the decrease of serine 32-phosphorylated IkB-*α* (Figure 3(b)) and total phosphorylated ERK 1/2 protein levels (Figure 3(c)), either after 30 min or 48 h. We then showed that 10^-8 ^M BNP treatment downregulated LPS/ATP-induced pro-IL-1*β* protein expression after 48 h (Figure 3(d)) exposure. Finally, we demonstrated NF-kB and ERK 1/2 involvement also in pro-IL-1*β* protein expression, finding that 10 *μ*M BAY 11-7082 (Figure 3(e)) and U0-126 (Figure 3(f)) pretreatment downregulated and reverted LPS/ATP-induced pro-IL-1*β* expression, respectively, after 48 h exposure. Altogether, these data demonstrated that BNP strongly inhibits the priming mechanism controlling IL-1*β* production by downregulating NF-kB and ERK/1/2 activation, both involved in pro- and mature IL-1*β* production.

### 3.4. BNP Is Involved in the Second Mechanism Controlling IL-1*β* Production by Inhibiting NALP3, ASC, and Caspase-1 Cascade Activation in THP-1 Cells

**Figure 4:**
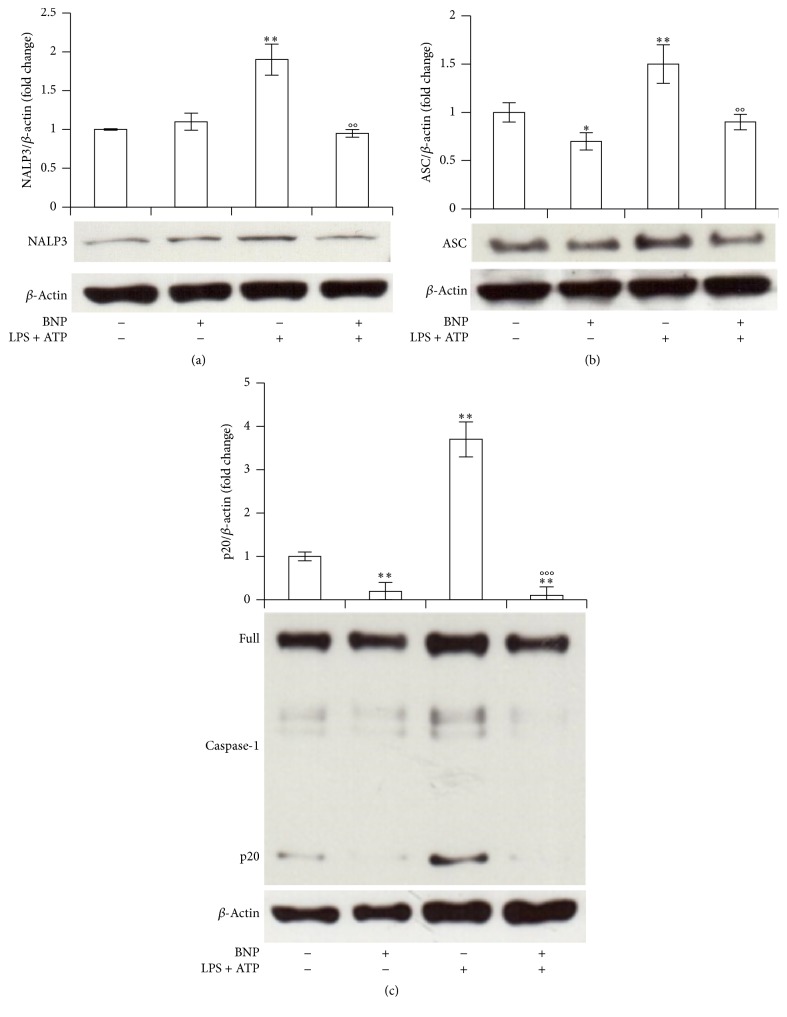
BNP is involved in the second mechanism controlling IL-1*β* production by inhibiting NALP3, ASC, and Caspase-1 cascade activation in THP-1 cells. THP-1 cells were treated with 10^−8 ^M BNP in absence or presence of 10 *μ*g/mL LPS/5 mM ATP for 30 min. Cell lysates were immunoblotted for NALP3 (a), ASC (b), or Caspase-1 (c). The blots were stripped of the bound Ab and reprobed with mouse anti-*β*-actin, to confirm equal loading. Western blots are representative of three separate experiments. Histograms indicate means ± SD of three separate experiments each one tested in triplicate. ^*∗*^*P* < 0.05 and ^*∗∗*^*P* < 0.01 versus untreated cells and °°*P* < 0.01 and °°°*P* < 0.001 versus LPS/ATP treated cells.

To evaluate BNP involvement also in the second signal driving the secretion of the active IL-1*β*, we investigated BNP effect on NALP3 cascade, the molecular platform whose activation triggers the maturation of Caspase-1, resulting in IL-1*β* production [1–4, 12]. We demonstrated that 10^-8 ^M BNP treatment abrogated 30 min LPS/ATP-induced NALP3 protein expression (Figure 4(a)), downregulated ASC protein expression, both at basal or after LPS/ATP stimulation (Figure 4(b)), and abrogated Caspase-1 activation, both at basal or after LPS/ATP stimulation, as indicated by the downregulation of the p20 active Caspase-1 fragment (Figure 4(c)). Altogether these results demonstrate BNP inhibitory action on the second part of the mechanism controlling IL-1*β* production.

### 3.5. NF-kB and ERK 1/2 Are Involved in NALP3/ASC/Caspase-1 Cascade Activation in THP-1 Cells

**Figure 5:**
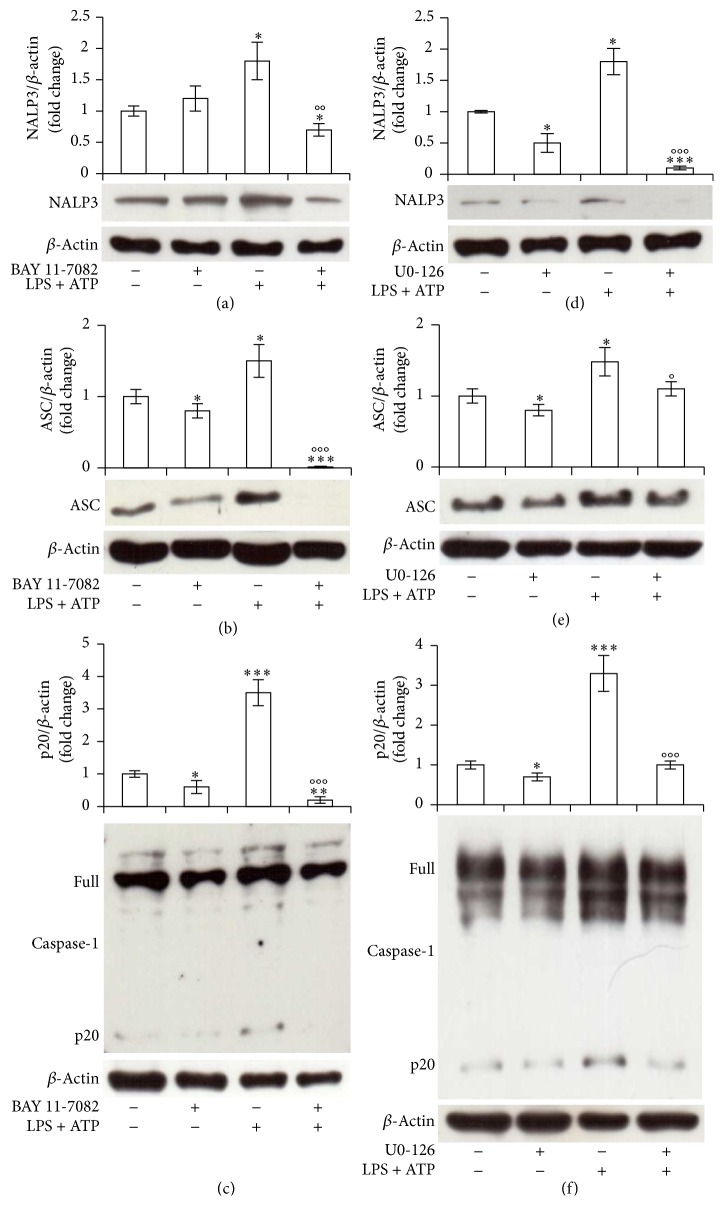
NF-kB and ERK 1/2 are involved in NALP3/ASC/Caspase-1 cascade activation in THP-1 cells. THP-1 cells were incubated with 10 *μ*M BAY 11-7082 (a, b, c) or U0-126 (d, e, f) for 1 h and then treated with 10 *μ*g/mL LPS/5 mM ATP for 30 min. Cell lysates were immunoblotted for NALP3 (a, d), ASC (b, e), or Caspase-1 (c, f). The blots were stripped of the bound Ab and reprobed with mouse anti-*β*-actin, to confirm equal loading. Western blots are representative of three separate experiments. Histograms indicate means ± SD of three separate experiments each one tested in triplicate. ^*∗*^*P* < 0.05, ^*∗∗*^*P* < 0.01, and ^*∗∗∗*^*P* < 0.001 versus untreated cells and °*P* < 0.05, °°*P* < 0.01, and °°°*P* < 0.001 versus LPS/ATP treated cells.

Both NF-kB and ERK 1/2 can be involved in NALP3 and Caspase-1 activation [4, 6, 46]. We then demonstrated the effective involvement of these pathways in our system, using the specific inhibitors BAY 11-7082 and U0-126. Ten *μ*M BAY 11-7082 or U0-126 1 h pretreatment, indeed, abrogated 30 min LPS/ATP-induced NALP3 (Figures 5(a) and 5(d)) or ASC (Figures 5(b) and 5(e)) expression and Caspase-1 activation (Figures 5(c) and 5(f)), showing the effective involvement of both pathways in all the elements of NALP/ASC/Caspase-1 cascade.

### 3.6. BNP Is Involved in All the Molecular Mechanisms Controlling IL-1*β* Production and Release by Inhibiting the Activation of NF-kB, ERK 1/2, and NALP3/ASC/Caspase-1 Cascade in Macrophage-Like Cells

**Figure 6:**
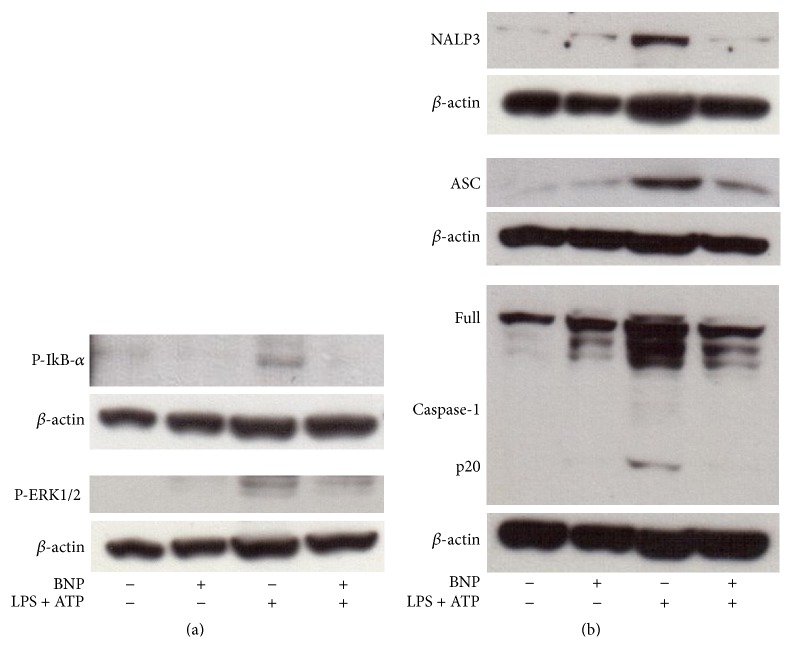
BNP is involved all the molecular mechanisms controlling IL-1*β* production and release by inhibiting the activation of NF-kB, ERK 1/2 and of all the elements of NALP3 inflammasome/ASC/Caspase-1 cascade in macrophage-like cells. PMA differentiated THP-1 cells were treated with 10^−8 ^M BNP in absence or presence of 10 *μ*g/mL LPS/5 mM ATP for 30 min. Cell lysates were immunoblotted for P-IkB-*α* and P-ERK1/2 (a) or NALP3, ASC, or Caspase-1 (b). The blots were stripped of the bound Ab and reprobed with mouse anti-*β*-actin, to confirm equal loading. Western blots are representative of three separate experiments.

BNP effect on the molecular mechanisms controlling IL-1*β* production and release was finally evaluated also in macrophages. By using PMA differentiated THP-1 cells, we demonstrated that 10^-8 ^M BNP treatment abrogated and downregulated 30 min LPS/ATP-induced NF-kB and ERK 1/2 activation, respectively (Figure 6(a)). Regarding BNP effect on NALP3 cascade, we demonstrated that 10^-8 ^M BNP treatment abrogated 30 min LPS/ATP-induced NALP3 protein expression and Caspase-1 activation and downregulated ASC protein expression, respectively (Figure 6(b)). Altogether these results demonstrate BNP inhibitory action on all the steps of the mechanism controlling IL-1*β* production and secretion also in macrophage-like cells.

## 4. Discussion

**Figure 7:**
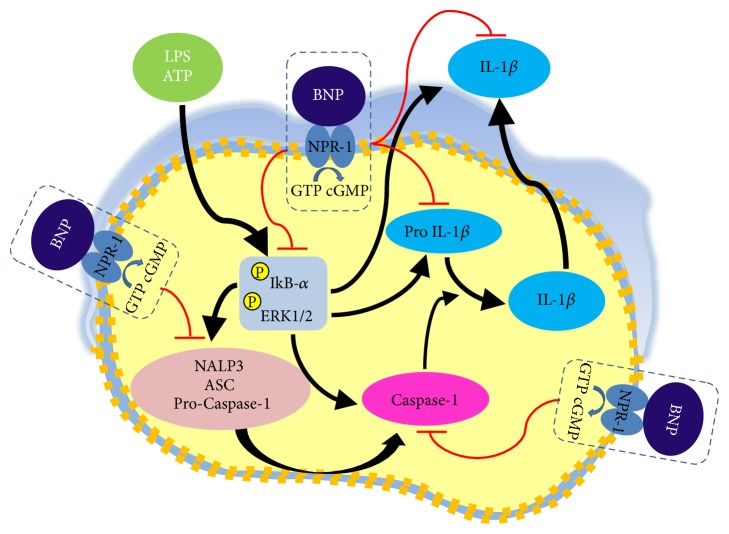
Mechanism of action of BNP inhibitory effect on LPS/ATP-induced IL-1*β* secretion in human THP-1 monocyte. BNP/NPR1/cGMP axis strongly inhibits IL-1*β* secretion by acting both at transcriptional and at posttranslational level. Firstly, by inhibiting the activation of NF-kB and ERK 1/2 signaling pathway, BNP downregulates the production of the inactive cytoplasmic precursor pro-IL-1*β*. Subsequently, again by inhibiting NF-kB and ERK 1/2 activation, BNP strongly downregulates all the elements of NALP3 inflammasome/ASC/Caspase-1 cascade and Caspase -1 activation, essential steps for the formation of the active secreted IL-1*β* protein.

IL-1*β* is a central mediator in inflammation and immunity. IL-1*β* production and release are tightly regulated by the activation of complex pathways such as NF-kB and ERK1/2, to induce pro-IL-1*β* upregulation, and NALP3/ASC/Caspase-1, to cleave pro-IL-1*β* into the biologically active secreted protein [4, 6, 12, 13, 46]. Considering the pathogenic roles of IL-1*β*, NF-kB, ERK1/2, and NALP3 inflammasome inappropriate activation in a large number of major human disorders and in cancer [1, 2, 4–11, 15–28], a large interest is focused on the identification of molecules able to target them, in order to develop new therapeutic agents. IL-1*β* is a potent regulator of BNP [29], a hormone/paracrine/autocrine factor mainly involved in body fluid and cardiovascular homeostasis, via the guanylyl cyclase NPR-1 [30, 31, 34]. An emerging role for BNP in inflammation and immune system has been suggested, even though it remains largely unexplored. Here, we newly demonstrated that, in human THP-1 monocytes, LPS/ATP-induced IL-1*β* secretion is strongly inhibited by BNP/NPR-1/cGMP axis, at all the molecular mechanisms that tightly control its production and release: NF-kB, ERK 1/2 and all the elements of NALP3/ASC/Caspase-1 cascade (Figure 7). Due to the pivotal role of IL-1*β* on inflammation and immunomodulation, finding that an endogenous molecule, such as BNP, is able to strongly downregulate its release is particularly important. Moreover, these data support the anti-inflammatory and immunomodulatory role of BNP on human monocytes.

Our results, in line with BNP modulatory effect on other inflammatory mediators [36], suggest that during inflammation, where a complex network of pleiotropic mediators from plasma or cells (including monocytes and macrophages) occurs, BNP, by inhibiting IL-1*β* secretion, can affect the injured tissues-related inflammatory reactions, contributing to restoring cytokines production balance. In fact, since IL-1*β* stimulates the production and release of additional cytokines, BNP, by inhibiting it, can play an important compensatory role. Besides, in conditions where IL-1*β* and other cytokines levels are increased, such as after myocardial infraction and during progression of heart failure [42], elevated BNP plasma levels have been found [43]. Moreover, BNP levels increase also in patients during inflammatory contexts such as severe sepsis or septic shock [37, 39], and in several inflammatory pathologies with or without cardiac dysfunction [37]. Since IL-1*β* is a strong regulator of BNP promoter [29], we suggest that the observed BNP increase during IL-1*β*-related inflammatory contexts might be due to the direct effect of this cytokine. Concomitantly, the here found BNP inhibition of IL-1*β* release might contribute also to balance and/or restore BNP secretion itself, by an autocrine/paracrine loop. In support, NPR-1 is expressed on human monocytes [35, 38], macrophages [44], monocyte-derived dendritic cells [45], and peripheral blood mononuclear cells [35]. In agreement, here we newly report that THP-1 cells respond to BNP with an increase in cGMP production, thus indicating NPR-1 activation. Therefore, we suggest that in human monocytes, by inhibiting IL-1*β*, the BNP/NPR-1/cGMP axis has an immune-regulatory and anti-inflammatory role. In addition, we suggest that BNP might exert these actions also in tissue microenvironment, where BNP autocrine/paracrine mechanisms may occur [31]. This appears to be of special interest also in the pathogenesis of cardiovascular diseases, including hypertrophy, myocardial infraction, and heart failure, where the inflammatory response takes place and BNP release is particularly elevated [31–33]. Analyzing the molecular mechanism of BNP inhibitory action on IL-1*β* secretion, we firstly demonstrated the involvement of NF-kB and ERK1/2 pathways in our system, their inhibitors being able to strongly downregulate LPS/ATP-induced pro- and IL-1*β* production. Subsequently, we demonstrated BNP's capability to inhibit both these pathways and pro-IL-1*β* protein expression. The observed BNP inhibitory action on NF-kB and ERK 1/2 activation in human monocytes has been confirmed also in macrophages-like cells, in agreement with similar results obtained in animal models [47, 48]. Regarding NF-kB, we demonstrated that BNP inhibited IkB-*α* phosphorylation, essential for the release of the active form of this transcription factor [15]. Even though we did not investigate BNP inhibitory mechanism on NF-kB and ERK 1/2 activation, we hypothesize that this might be related to multiple processes. For instance, BNP inhibition of IkB-*α* phosphorylation could be the result of an indirect mechanism, occurring via BNP-induced ERK1/2 downregulation, this latter being a pathway involved in NF-kB activation by IkB-*α* phosphorylation [18]. Interestingly, the here observed BNP inhibition of IkB-*α* phosphorylation could be implicated also in the inhibitory effect of BNP on ERK 1/2, being IKK family involved in both NF-kB and ERK1/2 activation [17]. In addition, BNP-induced ERK 1/2 inhibition might be ascribed also to the here found BNP-induced increase in cGMP levels. In fact, both increased cGMP levels [49] or cGMP-dependent kinases exert a direct downregulation of ERK 1/2 activation [50]. In addition, we do not exclude that both NF-kB or ERK 1/2 inhibitions may be the result of a negative feed-back loop via IL-1*β*, this cytokine being an activator of both these pathways [1, 15]. Finally, such inhibitions could be related to the here observed downregulatory effect of BNP on ASC expression, ASC itself being an important activator of both these pathways [2, 28]. The biological relevance of BNP-induced NF-kB/ERK1/2 inhibitions is that BNP may exert an anti-inflammatory and immunomodulatory role via the regulation of such pathways. In fact, both these signaling are involved in LPS-induced proinflammatory responses and their activation result in a rapid release of proinflammatory cytokines into the circulation, leading to an additional inflammatory response [15, 46]. In addition, it has been demonstrated in mouse macrophages that NF-kB activation additionally turns on NALP3 transcription, thereby licensing NALP3 inflammasome activation [6, 51]. Therefore, we suggest that BNP inhibitory effect on NF-kB and ERK 1/2 can be further investigated for a potential therapeutic employment of this hormone in the treatment of inflammatory diseases, where the application of chemical NF-kB and ERK 1/2 inhibitors may cause problems related to their efficacy and side effects [52, 53]. Besides, BNP infusion is used on top of standard care, providing clinical benefit in acutely decompensated heart failure patients [54, 55]. Regarding the second part of the mechanism controlling IL-1*β* secretion, we showed that BNP completely reverted LPS/ATP-induced NALP3, ASC and pro-Caspase-1 protein expression and NALP3 inflammasome activation both in monocytes and in macrophage-like cells. BNP capability to downregulate NALP3/ASC/Caspase-1 protein expression demonstrates its involvement in the priming/licensing step of inflammasome activation in these different cellular systems and represents an important new mechanism of action for this natriuretic peptide. In addition, we demonstrated that BNP completely blocked LPS/ATP-induced Caspase-1 active fragment production, both in THP-1 monocyte and in macrophage-like cells. Since the hallmark of NALP3 inflammasome activation is the proteolytic cleavage of Caspase-1 that, in turn, cleaves pro-IL-1*β* into the mature form IL-1*β,* our results indicate BNP involvement also in the second mechanism of inflammasome activation. This effect appears to be particularly important since NALP3 inflammasome components may have roles also in inflammasome-independent cytokine production and noninflammasome-related cytokines can influence the activation of the inflammasome [6]. Therefore, we suggest that BNP may exert anti-inflammatory and immunomodulatory effect also via the downregulation of both the elements of this molecular platform and its activation, implicated in the pathogenesis of major human diseases with a high prevalence worldwide [1, 4–11, 19–27]. Hence, BNP could be further investigated also for its potential therapeutic effect on NALP3/ASC/Caspase-1 associated disorders also in consideration of the fact that many of the autoimmune diseases are refractory to most therapies and that immunosuppressive agents have many side effects [6]. Analyzing the mechanism of action of BNP inhibitory effect on NALP3/ASC/Caspase-1 cascade, we finally demonstrated that it is directly related to BNP deregulatory effect on NF-kB and ERK 1/2 activation. In fact, the specific BAY 11-7082 and U0-126 inhibitors completely reverted LPS/ATP-induced NALP3, ASC, and Caspase-1 protein expression and Caspase-1 activation. These results are in agreement with previous data demonstrating that NALP3 expression is dependent on NF-kB activation [4, 51] and that BAY 11-7082 is able to inhibit both NALP3 inflammasome and Caspase-1 activation in a murine model [56]. Moreover, it has been recently demonstrated that NALP3 inflammasome activation by LPS requires ERK 1/2 and that U0-126 significantly blocks it [46].

## 5. Conclusions

In conclusion, we demonstrated a novel role for BNP/NPR-1/cGMP axis: downregulation of IL-1*β* secretion from THP-1 monocytes, via a strong inhibition of all the molecular mechanisms controlling its production and secretion: NF-kB, ERK 1/2, and NALP3/ASC/Caspase-1 cascade. Regarding BNP inhibitory effect on NALP3 inflammasome activation, we demonstrated that it is related to BNP-induced downregulation of NF-kB and ERK 1/2 activation.

Our data point out a potent anti-inflammatory and immunomodulatory role for this peptide suggesting a possible employment of this endogenous agent, already used for the treatment of heart failure [54, 55], also in the treatment of inflammatory/immune-related and IL-1*β*/NF-kB/ERK1/2/NALP3/ASC/Caspase-1-associated diseases, known to affect millions of people worldwide.
